# The manoeuvrability hypothesis to explain the maintenance of bilateral symmetry in animal evolution

**DOI:** 10.1186/1745-6150-7-22

**Published:** 2012-07-12

**Authors:** Gábor Holló, Mihály Novák

**Affiliations:** 1 Institute of Psychology, University of Debrecen, P.O.B. 28, Debrecen, H, 4010, Hungary; 2 Faculty of Applied Sciences, Campus du Solbosch, bâtiment U, Université Libre de Bruxelles, avenue F. D. Roosevelt 50, 1050, Brussels, Belgium; 3 Institute of Nuclear Research of the Hungarian Academy of Sciences (MTA ATOMKI), Debrecen, Hungary

**Keywords:** Bilateral symmetry, Radial symmetry, Manoeuvrability, Drag, Drag coefficient

## Abstract

**Background:**

The overwhelming majority of animal species exhibit bilateral symmetry. However, the precise evolutionary importance of bilateral symmetry is unknown, although elements of the understanding of the phenomenon have been present within the scientific community for decades.

**Presentation of the hypothesis:**

Here we show, with very simple physical laws, that locomotion in three-dimensional macro-world space is itself sufficient to explain the maintenance of bilateral symmetry in animal evolution. The ability to change direction, a key element of locomotion, requires the generation of instantaneous “pushing” surfaces, from which the animal can obtain the necessary force to depart in the new direction. We show that bilateral is the only type of symmetry that can maximize this force; thus, an actively locomoting bilateral body can have the maximal manoeuvrability as compared to other symmetry types. This confers an obvious selective advantage on the bilateral animal.

**Implications of the hypothesis:**

These considerations imply the view that animal evolution is a highly channelled process, in which bilateral and radial body symmetries seem to be inevitable.

**Reviewers:**

This article was reviewed by Gáspár Jékely, L. Aravind and Eugene Koonin.

## Background

Animals show diverse types of symmetry including spherical, cylindrical (also known as perfect radial), radial, biradial, bilateral and asymmetric (for review, see ref. [1]). In this paper, the terms cylindrical and radial will be used as synonyms because in the context it makes no substantial difference. More than 99 % of animal species are bilaterally symmetrical. The few exceptions are the amorphous parasitic placozoan *Trichoplax*, sponges (with asymmetry, spherical symmetry, and elements of radial symmetry in the skeleton), cnidarians (which include jellyfish, hydras, corals and sea anemones with radial and biradial symmetry), ctenophores or comb jellies (biradial symmetry), and echinoderms (sea lilies, sea urchins, and sea stars with radial symmetry).

Bilateral symmetry with two body axes arose early in animal evolution, probably in slow, flat, worm-like organisms locomoting on a substrate [2]. Genetic analyses have concluded that the genes responsible for bilateral symmetry most likely appeared prior to the cnidarian–bilaterian split [3-6], in the Precambrian [7,8]. However, even if the slow, cilium-based locomotion on a substrate may explain the generation of bilateral symmetry, it certainly cannot account for its survival over millions of years of animal evolution. Most recent animal species are bilaterally symmetrical, muscle-based locomoters, either living a pelagic life in water, or locomoting on the land and/or in the air. Why has bilaterality, which probably formed in benthic lifestyle, also proved so succesful in free-moving animals?

## Presentation of the hypothesis

Now we focus on the aquatic environment because bilateral symmetry (and animal life, itself) formed there, and had to be maintained there for millions of years, before bilaterians conquered the land. Let us start with the elementary physical fact that to locomote in a fluid, a body has to overcome drag (the resistance of the medium in which the body moves, acting in opposition to the direction of locomotion).

The magnitude of the drag force is:

*F = – ½ ρ c A v*^*2*^

where *F* is the drag force, *ρ* is the density of the medium, *c* is the dimensionless drag coefficient dependent on the body shape, *A* is the area of the maximal section of the body in the direction of motion, and *v* is the body’s velocity [9,10]. The negative sign on the right side indicates that drag is opposite to the direction of motion. It is important to note that this equation is valid for situations where the viscous forces are negligible compared to inertial forces, in what is loosely described as the macroscopic world (i.e at high Reynolds numbers). In the microscopic world, the forces are dominated by the viscosity of the fluid rather than by the inertia (i.e. at low Reynolds numbers) [10], however a discussion of the locomotion in the micro scale world is not the concern of this paper (for an in-depth analysis see ref. [11]).

Given the fact that the medium imposes resistance on the body, if resistance forces are unequally distributed around the body, their resultant force will not be zero compared to the rectilinear direction (i.e. movement straight ahead), so the body will not move on a linear path. This is the case when a moving body is asymmetric. Thus, it follows that a directionally locomoting animal has to be symmetric in order to avoid this effect. To be able to move forward, the animal can have any type of symmetry, so the approach outlined here is not sufficient to explain the success of bilateral symmetry. Rectilinear motion is, however, not the only element of locomotion. One other important element is changing direction, the importance of which, in this regard, has been mostly ignored in the literature so far. A slight deviation from the straight trajectory can easily be obtained by flawing one element of symmetry, thus generating asymmetry in the original direction of motion. This can be achieved by any symmetrical body. However, when a quick changeover is required, the situation becomes very different.

In quick changes in direction, the body has to exercise a force in the opposite direction to the desired new orientation. This means that it has to have a “pushing” surface in water from which to depart in the new direction. This surface is formed by the water layer against which the body is standing in order to push itself away, and it is produced by creating a great instantaneous drag force. Since *ρ* in the equation is unchanged, and *v* is diminishing or constant, the animal has to increase the maximal surface *A* and/or the drag coefficient *c*.

We will now overview the main symmetry types in terms of their capacity to create a pushing-off drag force. Given that a swimming body has to minimize the overall drag, its skin friction [9], and thus its wetted area, has to be adequately reduced. Thus, only three main body forms can be considered: spherical (with endless symmetry planes and symmetry axes), cylindrical (with endless symmetry planes and one symmetry axis) and bilateral (with one plane of symmetry). An elongated radial body that shows a star-like section is suboptimal since it has a very large surface that is far from ideal for swimming forwards.

A spherically symmetrical body cannot generate the pushing surface, being of equal shape and drag in every direction. Since the forces – which are different from the one operating in the direction of its motion – acting on this body are all equalized, it will not be able to depart in a new direction. It can only rotate around itself to deviate to a small extent (as soccer players bend the ball), but this is hardly an effective changeover and obviously cannot guarantee accurate manoeuvrability (understood simply as the capacity to perform quick and accurate changeovers). In this context we can disregard how it was able to move directly in the first place.

**Figure 1:**
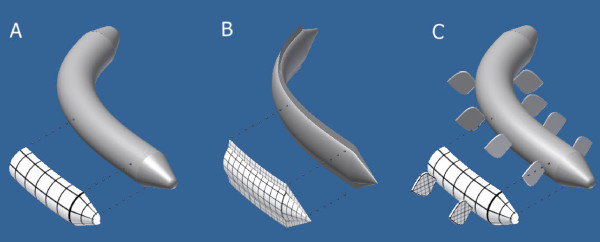
**Schematic representation of a cylindrical (A) and two bilateral bodies (B and C) generating pushing surfaces while changing direction.** Denser grids indicate a greater drag force.

A cylindrical (Figure 1.A) or approximately cylindrical (or radial) body locomoting with lateral or vertical undulation is able to increase *A*, which will be generated by a section of its body opposed to the direction in which it wants to move. The area of this surface is given approximately by the product of the diameter and the length of the body portion in question (and of course by its angular orientation to the axis of translation). If its lateral drag coefficient (*c*) is greater than the frontal, then when the animal turns its body can also increase *c* in the equation. However, regardless of the relationship between the anterior and lateral *c*, if the product (*c A*) from the lateral view is greater than that from the frontal one, this body will be able to move forward as well as to change direction.

A bilateral body (Figure 1.B and C) can alter both coefficients *A* and *c* as well. Since it has only one plane of symmetry (in the main direction of the motion), vertically it can carry structures with an extended surface area. The lateral area (*A*) of the body will be further increased by these structures (just think of the vertically posed fins of a shark). Furthermore, equipped with these, and with the more or less flattened sides of the body, the animal can also greatly increase *c*. Since it is streamlined only from the frontal view, its lateral (or vertical if the animal is dorsoventrally flattened) drag coefficient is very high compared to the frontal one.

The flatter its sides – including the appendages – are, the greater its lateral drag coefficient will be as compared to that of a cylindrical body. And knowing that a rectangular plate has an approximately 50 to 70 % higher drag coefficient (depending on the height to length ratio) than a cylinder (at Re = 10^5^) [9], we can say that bilateral symmetry offers the evolutionary possibility of increasing *F* by as much as 50 to 70 % compared to cylindrical symmetry, thanks simply to the drag coefficient. In other words, when a hypothetical cylindrical and a bilateral body have the same *A* (and frontal *c*), the bilateral body will enjoy a greater advantage in turning because it can produce a pushing force much greater than the cylindrical body because laterally it is less streamlined. Is this condition sufficient to assure a marked evolutionary advantage for bilateral symmetry? Since this capacity offers a very effective locomotion with potentially excellent manoeuvrability, we suggest that it is. Otherwise we would have to argue that effective locomotion is not a great advantage for an organism for whom a basic feature is precisely locomotion. Compared to a bilateral body, the cylindrical form has lower resistance in sideways movement, so the cylindrical body “slides” laterally in changeovers, as we do when we try to change direction on ice.

One could argue that a bilateral body can manoeuvre well only in left-right directions while a cylindrical body can, in theory, turn in every direction away from that of the motion. Bilateral animals, being not rigid objects (like ships or aircraft are), solve the problem simply by twisting the body and the appendages in the desired directions.

Based on the arguments explained so far it could be stated that a symmetry that is streamlined in only one direction, while non-streamlined in other directions, is favourable for manoeuvrable locomotion.

It is important to say that the changeover does not necessarily have to be drag-assisted. Some radially symmetrical animals, such as jellyfish, use asymmetric contractions of the bell, thus generating asymmetric jet flows to steer. However, the accuracy and the speed of this medusan-type manoeuvring [12] are much more modest than the drag-based manoeuvring of bilateral pelagic animals.

Bilateral symmetry has also proved to be succesful both on land and in the air. On land, the force-generating role of the drag in water is replaced by gravitation and so by the necessity of leaning on the land. In this regard, locomotion on land is analogous to that on the fluid–solid interface. This locomotion essentially occurs in two dimensions, thus, direction shift on land requires the body to be capable of turning left or right, and so of being supported from the right as well as from the left. The effectiveness of creeping locomotion has been improved by the evolution of limbs, which, placed on the two sides of the bilateral body, satisfy the above-mentioned condition. (For the sake of simplicity, we will not deal with the limbless evolution of snakes and limbless lizards here.)

Flying, similarly to swimming, requires the animal to create pushing surfaces in the air. The evolution of large-surface wings allowed the animals to locomote in a medium which, compared to water, has a lower density, and as a consequence, is almost completely lacking in the hydrostatic pressure that to a certain extent counterbalances the force of gravity in water.

The combination of bilaterality with the centralisation of the nervous system and cephalisation allowed the evolution of really successful body plans ensuring precise locomotion and rapid information processing.

## Implications of the hypothesis

### Radial symmetry

From the principles developed so far it follows that asymmetry or radial symmetry could have evolved only in animals which do not locomote or locomote slowly. (We use the term ‘slow’ intuitively because an exact speed limit depends on the animal’s mass, form, and size as well as on the mode of locomotion and on the speed of water flow around it. Hence it would vary from species to species. We hope that the understanding of the essence of this concept will not be disturbed by the absence of a clearly defined value.) By sacrificing quick locomotion, these animals necessarily become more vulnerable to predators. Thus they have to be well protected (e.g. echinoderms, cnidarians), and this protection can be further strengthened by being of very low nutritive value (sponges, ctenophores, cnidarians).

The radial body symmetry will be ideal for these animals because it confers on the body the ability to react to environmental forces in every direction (sessile cnidarians and echinoderms), to be able to catch food around with the same probability (cnidarians, ctenophores, echinoderms) and to maintain a static position, adhering to the substratum against water currents (locomoting echinoderms) [1,10,13].

There is also another evolutionary situation in which the body has to be externally cylindrical: a burrowing lifestyle. This lifestyle develops when an animal lives and locomotes in a very dense medium: in earth or equivalents and in the body of other organisms. In these media the density is so high that any lateral structures which increase surface area will be disadvantageous. So the external body form will be the one that assures a minimum cross section and hence a minimum friction per body mass; and at the same time also reduces the vulnerable body surface. This form is cylindrical. Naturally, this does not necessarily mean that externally bilateral burrowing animals cannot exist but, even in this case, their main body form tends to be nearly cylindrical and the surface-augmenting effects of limbs must be counterbalanced by their burrowing or other functions (e.g. subterranean rodents; ref. [14]).

Here it is crucial to note that animal body symmetry is often different externally and internally [1,3]. It is enough to consider that the external side of the animal interacts directly with the environment while the internal side does not. Hence they face very different conditions that may require different symmetries.

### Apparent problems with the association of symmetry and locomotion

Since those animals which are not bilaterally symmetrical are typically sessile or planctonic drifters, while most bilaterals are free locomoting, the association of bilateral symmetry with directed locomotion seems obvious. Beklemishev [2] pointed out that when the body is asymmetric, as it reaches a certain speed, rectilinear locomotion becomes impossible and the body begins to move in a helical trajectory. As he explains, the advantage of bilateral symmetry is precisely that the environmental pressures on the two sides of the body are equalized, guaranteeing a rectilinear locomotion. Following this view, the close association between free swimming and bilaterality has also become widespread in textbooks (e.g. refs. [15-18]). However, it could also be due to the lack of an adequate explanation for this – otherwise widely accepted – relationship that several authors have questioned it. It has been hypothesized that the origin of bilateral symmetry in animals could have been favoured by internal transport, not by directed locomotion [19]. Based partly on this view, it seemed problematic to couple the tetraradial symmetry and the active locomotion of the endoparasite cnidarian *Buddenbrockia*, so a further dissociation of symmetry from locomotion has been proposed [20]. It has also been reported that the bilateral body form [21] and the bilateral spine distribution [22] of sea urchin species was connected to efficient body protection, not to efficient locomotion.

Based on the concept presented here it can be understood that the cylindrical external form and the internal tetraradiality of *Buddenbrockia* is not inconsistent with its active locomotion [20], and that the slow locomotion of a sea urchin does not have to be closely related to its bilateral body form [21] or its bilateral spine distribution [22].

Another potential question may emerge if one examines the earliest trace fossils from the Precambrian. These traces are retained horizontal burrowings in the upper layer of the sediment [23,24] and are also attributed to bilaterian animals [24]. However, this view has been challenged by the discovery of trace maker giant protists [25], put forward as candidates for the producers of those ancient trails. Now, according to our hypothesis, it seems easy to reconcile the putative burrowing behaviour and bilaterality in the precambrian animals mentioned above (if they really existed) considering that the upper layer of the sediment is likely to have a loose structure with low density, hence it does not necessarily require the body burrowing in it to be cylindrical.

## Conclusions

It has been suggested that radial and bilateral body plans could have been generated with the same or similar genetic toolkit but with different regulatory networks [8,26-28]. This means that most likely there was no genetic barrier to the emergence and evolutionary competition of the two body plans. Whatever the case, we argue that this competition was strongly determined by the physical laws of locomotion.

Here, we do not consider the temporal priority of radial or bilateral symmetry in early animal evolution (but see refs. [1,5,6,19,28,29]), and similarly, we do not take a stand on the lifestyle of the first bilaterians [8,19,30,31]. We only state that, from the moment bilateral symmetry arose in macro-animal evolution it represented a potentially enormous selective advantage over other body plans assuring faster changeovers and a more precisely directed locomotion. This is a key to survival both for prey and for predators.

At the system level, these considerations mean animal evolution should be viewed as a strongly channelled process (cf. ref. [32]) – a product of which is an enormous variation of bilateral organisms rather than “endless forms” [33] of living systems.

Very probably, other key selective forces also influenced and influence the evolution of basic animal body plans. However, these factors are yet to be explored. We propose one of them – one that may in itself be enough to favour bilateral symmetry against other symmetries.

## Competing interests

The authors declare that they have no competing interests.

## Authors’ contributions

G.H. conceived the idea and formulated the main lines of the theory. M.N. pointed out the importance of changeover in locomotion and provided important elements of the simplest physical explanation. G.H. elaborated the final version of the theory and wrote the paper. All authors read and approved the final manuscript.

## Reviewers’ comments

Reviewer 1: Gáspár Jékely

This is an interesting analysis providing a physical explanation for the maintenance of bilateral symmetry in animal evolution. I find the paper well written, the arguments convincing, and only have a few comments to clarify the discussion.

The authors refrain from discussing locomotion in the microscopic world. However, I think that they miss an opportunity here. We know that in the micro world many organisms can navigate very efficiently. They achieved this not by being bilaterally symmetrical, but by using helical swimming and the adjustment of the helical trajectories. This happens very often in diverse phototactic protist (e.g. Chlamydomonas, dinoflagellates) and in the close-to spherical ciliated larvae of bilaterians (e.g. annelids, hemichordates). One reason why this is an effective strategy for small organisms but not large ones is, as discussed in the paper, the different Reynolds numbers. This could imply that bilaterality only evolved once the early metazoans had attained a sufficiently large size. This interesting physical threshold could be discussed in more detail.

Authors’ response: *First of all we thank Dr. Jékely for his invaluable work. The idea that bilaterality evolved when the animals at hand had reached a certain size (e.g. Valentine Proc Natl Acad Sci USA 1994,****91:****6751–6757) cannot be proved by convincing evidence at present (see for example Chen et al. Science,****305:****218–222 – although this is controversial [e.g. Chapter 1 by Budd GE in Animal Evolution: Genomes, Fossils, and Trees edited by Telford MJ, Littlewood DTJ, 2009]). The fact that several small animals, living in the realm of low Reynolds numbers, have bilateral symmetry probably indicates that bilaterality could have evolved in the microscopic world, but most likely it does not offer the kind of advantage over other symmetries there as it does in the macro world. Until it can be excluded that certain factors could favour bilateral symmetry in the micro world, we would rather avoid taking a stand on this – otherwise exciting – evolutionary problem. Please see also response Nr. 1 to Dr. Aravind.*

The model implies that the first bilaterians were not burrowing but either freely swimming or crawling on the sediment. It would be interesting to see a discussion of this in the context of the earliest putatively bilaterian trace fossils. The interpretation that these are traces of burrowing animals may be slightly at odds with the hypothesis (e.g. Jensen Integr. Comp. Biol., 43:219–228).

Authors’ response: *Given that the precise origin of the first bilaterians is unproven we would not like to take sides on their lifestyle; nevertheless, the topic of the trace fossils, still highly controversial, is very interesting. The following paragraph has been added to the “Apparent problems with the association of symmetry and locomotion” section:*

“Another potential question may emerge if one examines the earliest trace fossils from the Precambrian. These traces are retained horizontal burrowings in the upper layer of the sediment [23,24] and are also attributed to bilaterian animals [24]. However, this view has been challenged by the discovery of trace maker giant protists [25], put forward as candidates for the producers of those ancient trails. Now, according to our hypothesis, it seems easy to reconcile the putative burrowing behaviour and bilaterality in the precambrian animals mentioned above (if they really existed) considering that the upper layer of the sediment is likely to have a loose structure with low density, hence it does not necessarily require the body burrowing in it to be cylindrical.”

Accordingly, the cited references also have been added to the paper.

I suggest to also include in Figure 1. a bilaterian with a cylindrical body but with lateral appendages. A laterally flattened, fish-like body is not general for actively moving bilaterians. For example, errant annelid polychaetes have a body that is roughly cylindrical, but they have lateral appendages that can provide the necessary drag during active locomotion.

Authors’ response: *The figure has been added as Figure 1.C.*

The authors use the term ‘aerodynamic’ when writing about locomotion primarily in water. Using ‘drag’ or ‘resistance’ may be more fortunate.

Authors’ response: *The word “aerodynamic” has been replaced by more appropriate ones.*

The sentences in question now read: “… where *F* is the drag force, *ρ* is the density of the medium, *c* is the dimensionless drag coefficient dependent on the body shape, *A* is the area of the maximal section of the body in the direction of motion, and *v* is the body’s velocity [9,10].”

“A spherically symmetrical body cannot generate the pushing surface, being of equal shape and drag in every direction.”

“Since it is streamlined only from the frontal view, its lateral (or vertical if the animal is dorsoventrally flattened) drag coefficient is very high compared to the frontal one.”

“Compared to a bilateral body, the cylindrical form has lower resistance in sideways movement, so the cylindrical body “slides” laterally in changeovers, as we do when we try to change direction on ice.”

The statement that “Bilateral symmetry with two body axes arose … in slow, flatworm-like organisms” together with Ref. [7] seems to imply that the first bilaterians were phylogenetically related to acoel flatworms. The latest careful phylogenetic analyses show that acoels are deuterostomes (Nature 470, 255–258), so they do not represent the earliest extant bilaterian metazoans. I suggest to write ‘worm-like organisms’.

Authors’ response: *It has been changed to “flat, worm-like organisms”; remaining, at the same time, preferably faithful to the cited reference.*

A reference to jellyfish navigation (e.g. Garm et al. The Journal of Experimental Biology 210, 3616–3623) would make the discussion about the medusa-type manoeuvering more convincing.

Authors’ response: *Thank you, the reference has been included.*

Reviewer 2: L. Aravind

Since Beklemishev it has been generally accepted among students of zoology that the advantages of directed movements are the driving force for the origin and maintenance of bilateral symmetry, the dominant form of symmetry among metazoans. It is usually imagined that such this symmetry emerged in benthic contexts – creeping on a substratum enable favored dorso-ventral differentiation, which coupled with selection for effective directed movement resulted in a bilateral form. However, this has been questioned on the basis of the observations of the asymmetric expression of TGF-beta family members and Short gastrulation orthologs along the directive axis in cnidarians. This implies that even if the ancestral metazoan was outwardly radial symmetric, there might have been a pre-adaptation or pre-disposition for bilaterality as suggested by the situation in cnidarians. This has been used by Finnerty to argue for a role for internal circulation within the gut lumen as a major factor in the origin of bilateral symmetry. The molecular evidence on the whole favors a single major origin for bilaterality in animals, but is subsequent strong maintenance remains less explained.

Here the authors present a simple physical explanation as to why bilaterality is a more stable strategy than any other symmetry once directed locomotion emerges. Central to their explanation is its role in maneuverability that has apparently not been used as done by the authors in this article. The physical arguments by the authors point to bilaterality being an apparently stable strategy at large Reynolds number where the equation used by them for drag forces is appropriate.

However, the main question that arises how large should be the Reynolds number be for this argument to hold. Looking up these values it appears that an unicellular free-swimming eukaryotes might have Re = 10^-1. Here the viscous forces are probably dominant, allowing for the asymmetric morphology of such forms, e.g. ciliates and dinoflagellates. The smallest vertebrate is said to have Re ~ 1 and bilateral metazoans like chaetognatha and rotifer have Re in between those figures. It should be noted that they have strongly bilateral forms. Further, the estimate sizes for the basal bilateralians do not place them much higher than this range in terms of Re. So the key question that arises is whether at these sizes the argument based on negligible viscous forces is entirely valid. It would be good for the authors to consider this issue and present potential tests for their hypothesis.

Authors’ response: *We thank Dr. Aravind for his valuable work. The fact that bilateral symmetry is also present in the environment of low Reynolds numbers does not necessarily contradict our hypothesis. The very different pattern of main body symmetries between low and high Re environments probably emerges from their different relations to viscous and inertial forces. But this difference does not necessarily exclude bilateral symmetry from the small-scale world – although manifestly it does not enjoy the advantage over other symmetries which it does in the large-scale world. Our hypothesis implies that bilateral symmetry is advantageous in the high Re-world but this does not mean bilaterality could not have been favoured by certain factors in the low Re-world. However, these factors – to our knowledge – have not been clarified since Beklemishev. Please see also response Nr. 1 to Dr. Jékely.*

Minor points

<<Bilateral symmetry with two body axes arose early in animal evolution, probably in slow flatworm-like organisms locomoting on a substrate [2], likely prior to the Cnidarian–Bilaterian split [3-6] in the Precambrian [7,8].>>

This sentence is potentially confusing, because it might present a contradiction within it. The authors need to clarify as to what they mean by origin of bilateral symmetry prior to the origin of Bilateria (i.e. the flatworm like organisms). Are they meaning the situation in cnidarians or reconstructing some ancestral form?

Authors’ response: *Thank you, this part has hopefully been clarified, and it reads now:*

“Bilateral symmetry with two body axes arose early in animal evolution, probably in slow, flat, worm-like organisms locomoting on a substrate [2]. Genetic analyses have concluded that the genes responsible for bilateral symmetry most likely appeared prior to the cnidarian–bilaterian split [3-6], in the Precambrian [7,8].” <<“a symmetry that is streamlined in only one direction, while non-aerodynamic in other directions, is favourable for locomotion.” >>

May be the last word should be replaced with maneuverable locomotion.

Authors’ response: *The word “manoeuvrable” has been inserted in the sentence, thank you:*

“a symmetry that is streamlined in only one direction, while non-aerodynamic in other directions, is favourable for manoeuvrable locomotion.”

Reviewer 3: Eugene Koonin

The authors of this manuscript strive to provide an explanation for the domination of bilateral symmetry in animals. The come up with the idea that bilateral symmetry provides for by far greater ability to swiftly change the direction of movement than any other body plan, hence a substantial advantage for free moving animals. The authors submit that this is a major factor behind the near ubiquity of bilateral symmetry but they are careful in indicating that other factors could be important as well. In my view, this is an interesting and sensible hypothesis although I think that it would gain in strength should the authors coach their hypothesis in specific equations of mechanics.

Authors’ response: *We thank Dr. Koonin for taking charge of the review of the manuscript. While formulating the hypothesis we constantly endeavoured to provide the simplest explanation for the problem in the clearest way. We think the basic statements (e.g. the body has to overcome drag; to push itself in a new direction it has to exercise a force in the opposite direction) and the equation of drag with some other minor considerations are necessary and sufficient arguments for the explanation of the theory, but should it require a more detailed rationale we would be grateful for more specific instructions. Please also consider that this is the main hypothesis that may serve as a basis for more specific future analyses for particular scenarios.*
